# TGF-β1 increases proliferation of airway smooth muscle cells by phosphorylation of map kinases

**DOI:** 10.1186/1465-9921-7-2

**Published:** 2006-01-03

**Authors:** Gang Chen, Nasreen Khalil

**Affiliations:** 1 Division of Respiratory Medicine, Department of Medicine, The University of British Columbia and the Vancouver Coastal Health Research Institute, Vancouver, BC V6H 3Z6, Canada

## Abstract

**Background:**

Airway remodeling in asthma is the result of increased expression of connective tissue proteins, airway smooth muscle cell (ASMC) hyperplasia and hypertrophy. TGF-β1 has been found to increase ASMC proliferation. The activation of mitogen-activated protein kinases (MAPKs), p38, ERK, and JNK, is critical to the signal transduction associated with cell proliferation. In the present study, we determined the role of phosphorylated MAPKs in TGF-β1 induced ASMC proliferation.

**Methods:**

Confluent and growth-arrested bovine ASMCs were treated with TGF-β1. Proliferation was measured by [^3^H]-thymidine incorporation and cell counting. Expressions of phosphorylated p38, ERK1/2, and JNK were determined by Western analysis.

**Results:**

In a concentration-dependent manner, TGF-β1 increased [^3^H]-thymidine incorporation and cell number of ASMCs. TGF-β1 also enhanced serum-induced ASMC proliferation. Although ASMCs cultured with TGF-β1 had a significant increase in phosphorylated p38, ERK1/2, and JNK, the maximal phosphorylation of each MAPK had a varied onset after incubation with TGF-β1. TGF-β1 induced DNA synthesis was inhibited by SB 203580 or PD 98059, selective inhibitors of p38 and MAP kinase kinase (MEK), respectively. Antibodies against EGF, FGF-2, IGF-I, and PDGF did not inhibit the TGF-β1 induced DNA synthesis.

**Conclusion:**

Our data indicate that ASMCs proliferate in response to TGF-β1, which is mediated by phosphorylation of p38 and ERK1/2. These findings suggest that TGF-β1 which is expressed in airways of asthmatics may contribute to irreversible airway remodeling by enhancing ASMC proliferation.

## Introduction

Asthma is characterized by airway inflammation, hyperresponsiveness, and remodeling [1-3]. Severe asthmatics develop irreversible airway obstruction, which may be a consequence of persistent structural changes including increased airway smooth muscle cell (ASMC) mass in the airway wall that may be due to frequent stimulation of ASMCs by contractile agonists, inflammatory mediators, and growth factors [2,4]. Based on observations made on the pathogenesis of hyperproliferation at other sites, it is speculated that a number of cytokines may be important in regulating the proliferation of ASMCs. Of these cytokines, transforming growth factor-beta1 (TGF-β1), a multifunctional polypeptide, is one of the most potent regulators of connective tissue synthesis and cell proliferation [2,5-8].

The source of TGF-β1 in the airways may be from the inflammatory cells recruited to the airways or from the residential airway cells themselves such as bronchial epithelial cells and ASMCs [7,8]. We had previously demonstrated that all isoforms of TGF-β, as well as TGF-β receptor (TβR) type I and II were expressed by ASMCs in human and rat lungs [9,10]. In addition, we had found that in models emulating airway injury, such as *in vitro *wounding of confluent monolayers [11,12], exposure to proteases [12,13], or cells in subconfluent conditions [12], ASMCs released biologically active TGF-β1, which in turn led to increase in connective tissue proteins such as collagen I and fibronectin. Recently, we had reported that granulocyte macrophage-colony stimulating factor (GM-CSF), another cytokine found in asthmatic airways, increased connective tissue expression of bovine ASMCs in response to TGF-β1 by induction of TβRs [14]. TGF-β1 is likely to play an important role in airway remodeling in asthmatics. For example, Minshall et al [5] demonstrated that, as compared with the control subjects, both the expression of TGF-β1 mRNA and TGF-β1 immunoreactivity were increased in the airway submucous eosinophils, the cell that had been confirmed the presence of active TGF-β1, and these increases were directly related to the severity of the disorder. In a mouse model of airway remodeling induced by OVA sensitization and challenge, increased TGF-β1 was demonstrated by ELISA and immunohistochemistry with increased peribronchial collagen synthesis, thickness of peribronchial smooth muscle layer, and α-smooth muscle actin immunostaining [15]. Redington et al [6] found an increased TGF-β1 level in the bronchoalveolar lavage fluid from asthmatic patients compared to normal controls. Recently, McMillan et al [16] demonstrated that anti-TGF-β antibody significantly reduced peribronchiolar extracellular matrix deposition, ASMC proliferation, and mucus production in an allergen induced murine asthma model.

The effects of TGF-β1 on cell proliferation are more complex and context dependent [17,18]. For example, TGF-β1 inhibits proliferation of epithelial and hematopoietic cells [19]; however, TGF-β1 induces proliferation of the mesenchymal phenotype of cells such as fibroblasts, smooth muscle cells, and myofibroblasts [20]. Even within mesenchymal cells, the cell responses to TGF-β1 are highly variable. For example, TGF-β1 stimulates proliferation of confluent vascular and airway smooth muscle cells, but inhibits the proliferation of the same cells when they are subconfluent [21-24]. A low dose of TGF-β1 stimulates proliferation of fibroblasts, chondrocytes, and arterial smooth muscle cells, but a high dose of TGF-β1 inhibits the proliferation of the same cells [20,25]. The duration of TGF-β1 treatment also affects the cellular proliferative response to TGF-β1. For example, Incubation of ASMCs or articular chondrocytes for 24 hours with TGF-β1 inhibited cell proliferation, whereas 48- or 72-hour incubation stimulates proliferation of the same cells [26,27].

The proliferation of several phenotypes of cells is mediated by growth factor or cytokine induced mitogen-activated protein kinases (MAPKs), a family of serine-threonine protein. MAPKs consist of extracellular signal-regulated kinase (ERK), p38 MAPK (p38), and c-Jun NH_2_-terminal kinase (JNK) [28]. The activation of MAPKs is a key component in signal transduction associated with cell proliferation [29]. Among the three MAPKs, ERK has been well studied and proven to play a major role in the signalling of ASMC proliferation [30-38]. The activation of ERK by various substances, such as epidermal growth factor (EGF), platelet-derived growth factor (PDGF), fibroblast growth factor-2 (FGF-2, also called basic fibroblast growth factor, bFGF), insulin-like growth factor-I (IGF-I), thrombin, endothelin, phorbol esters, beta-hexosaminidase A (an endogenous mannosyl-rich glycoprotein), and 5-hydroxytryptamine (5-HT), increased ASMC proliferation [30-38]. The inhibitors or antisense oligonucleotide of ERK blocked the proliferation induced by these substances [30-37]. Activated ERK stimulates numerous transcription factors such as Elk-1, c-Jun, c-Fos, and c-Myc in the nucleus. The transcription factors in turn regulate the expression of genes required for DNA synthesis, such as cyclin D1. It has been demonstrated that active Ras and MAPK/ ERK kinase-1 (MEK1) (the upstream activator of ERK) each induced cyclin D1 promoter activity [36]. Elk-1 and activator protein-1 (c-Jun, c-Fos) reporter activation by mitogens was reduced by inhibition of MEK in human ASMCs [31]. In addition, inhibition of MEK attenuates mitogen-induced increase in promoter activity, mRNA or protein of cyclin D1 or c-Fos [30,32,38]. However, the role of p38 and JNK in mitogen-induced ASM proliferation is not well known. In addition, little is known about the role of MAPKs in TGF-β1 induced proliferation in ASMCs.

This study was designed to investigate the effect of TGF-β1 on asmc proliferation and the role of mapks in the TGF-β1 induced changes of asmc proliferation. We found that TGF-β1 increased asmc proliferation and the proliferative effects were mediated by phosphorylation of ERK1/2 and p38.

## Materials and methods

### Cell culture

Bovine trachea was obtained from a local slaughterhouse. An explanted culture of the smooth muscle tissue was established as described previously with some modification [14]. Briefly, the associated fat and connective tissues were removed in cold phosphate buffered saline (PBS) with antibiotic reagents (penicillin G 100 U/ml, streptomycin 100 μg/ml) and antimycotic reagent (amphotericin B 0.25 μg /ml). Then, the smooth muscle was isolated, cut into 1–2 mm cubic size, and placed on culture dishes with Dulbecco's modified Eagle's Medium (DMEM) supplemented with 10% fetal bovine serum (FBS) and antibiotic-antimycotic reagents. In an incubator at 37°C with a humidified atmosphere (5% CO_2_-balanced air), ASMCs migrated from the tissue explants and approached confluence around the explants. The explanted tissue was removed, and the ASMCs remaining in the culture were passaged with 0.05% trypsin/0.53 mM EDTA. Smooth muscle cell identity was verified by phase contrast microscopy for appearance of "hill and valley formation" and by immunocytochemistry staining for α-smooth muscle actin and smooth muscle-specific myosin heavy chain (SM1 and SM2). For the experiments, the ASMCs in passage 1–5 were plated at density of 10000 cells/cm^2 ^in DMEM with 10% FBS and antibiotic reagents. All reagents above were from GIBCO BRL (Burlington, ON, Canada). The cell viability was determined with trypan blue (Sigma, St. Louis, Missouri) exclusion.

**Figure 1:**
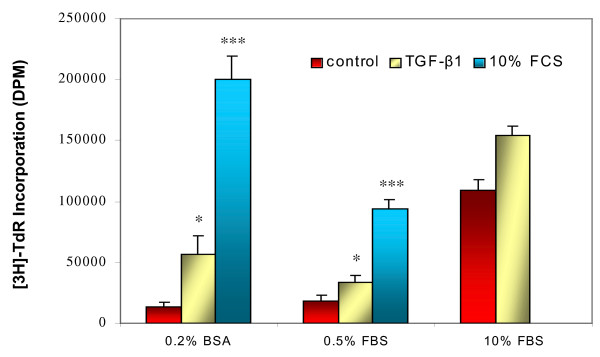
**ASMC responses to TGF-β1 and serum in different culture conditions. **ASMCs were cultured with DMEM/10% FBS to confluence and then changed to DMEM/0.2% BSA, DMEM/0.5% FBS, or DMEM/10% FBS for 72 hours, followed by treatment with 5 ng/ml of TGF-β1 or 10% FBS for 24 hours prior to [^3^H]-thymidine incorporation assay. * p < 0.05, *** p < 0.001 compared to control of the same condition. n = 4–6.

Since previous studies reported varied responses of TGF-β1 on ASMCs, we first determined an optimal culture condition for conducting the experiments. ASMCs were cultured in 24-well plates in DMEM with 10% FBS to confluence. After being washed with DMEM, the ASMCs were cultured for three days in one of following three media: DMEM with 0.2% bovine serum albumin (BSA, from Fisher Scientific, Fair Lawn, NJ), DMEM with 0.5% FBS, and DMEM with 10% FBS. Then, the cells were treated with 5 ng/ml of TGF-β1 (R&D Systems, Minneapolis, MN) or 10% FBS in the same fresh medium for 1 day followed by [^3^H]-thymidine incorporation and cell counting. As shown in Figure 1, increases in [^3^H]-thymidine incorporation occurred in all three conditions, but TGF-β1 and 10% FBS induced the strongest response in ASMCs cultured in 0.2% BSA/DMEM. Similar results were also seen in the number of cells (data not shown). Therefore, we chose 0.2% BSA/DMEM as the serum-free medium culture condition in which all further experiments were performed.

### Cell proliferation study

This study was performed by [^3^H]-thymidine incorporation and cell counting. Growth-arrested ASMCs were treated in serum-free medium in 24-well plates. Then, for some plates, [^3^H]-thymidine (1 μCi/ml, from ICN, Irvine, CA) was added for the final 4 hours and the incorporation was terminated by washing the cells with PBS twice. The cells were lysed with 0.2 N NaOH and the radioactivity was counted with a scintillation counter (Beckman LS5000CE). For other plates, the cells were washed with PBS, trypsinized and counted with a hemacytometer. To confirm the involvement of MAPKs in TGF-β1 induced proliferation of ASMCs, the cells were pretreated for one hour with 10 μM of SB 203580, 50 μM of PD 98059, or 10 μM of SP 600125, selective inhibitors of p38, MAP kinase kinase (MEK, which is upstream from ERK) and JNK, respectively (all from Calbiochem, San Diego, CA). Then 1 ng/ml of TGF-β1 was added to the medium and the cells were cultured for 24 hours, followed by [^3^H]-thymidine incorporation assay.

### Western blotting and immune detection

After treatment, ASMCs were washed with cold PBS and detached by trypsin. Whole cell protein was extracted on ice with lysis buffer (50 mM Tris-HCl pH 8.0, 0.15 M NaCl, 1% Triton-X-100, 0.1% SDS, 5 mg/ml sodium deoxycholate) in the presence of the protease inhibitors (as mentioned above) as well as phosphatase inhibitors including 1 mM NaF and 1 mM Na_3_VO_4 _(Sigma). Protein concentration was measured using the Bradford method with a BioRad Protein Assay Reagent (BioRad; Hercules, CA). Protein extracts were separated by SDS-PAGE on polyacrylamide SDS gels and then transferred onto a PVDF membrane (BioRad) as per Laemmli's method. After blockade with 5% milk in Tris-buffered saline containing 0.05% Tween-20, the membranes were incubated overnight at 4°C with following primary antibodies (from Cell Signaling, Beverly, MA): anti-total or anti-phosphorylated p38, ERK1/2 (which recognizes p42 and p44 MAPK), and JNK (which recognizes p46 and p54 JNK). This was followed by incubating the blot with a HRP-conjugated secondary antibody (Santa Cruz) for 1 hour at room temperature. The target proteins on the membrane were then immunodetected by the ECL system (Amersham, Arlington Heights, IL) according to the manufacturer's instruction. The equal loading of proteins was confirmed by immunodetecting the blots with anti-β-actin antibody (Sigma). Relative absorbance of the resultant bands was determined using the Quantity One imaging system (BioRad).

### Statistical analysis

The results were expressed as mean ± standard error of the mean (SEM). Student's t test and Kruskal-Wallis test combined with Dwass-Steel-Chritchlow-Fligner test were used for the data analysis. Differences were considered statistically significant when p < 0.05.

## Results

### TGF-β1 increased ASMC proliferation

**Figure 2:**
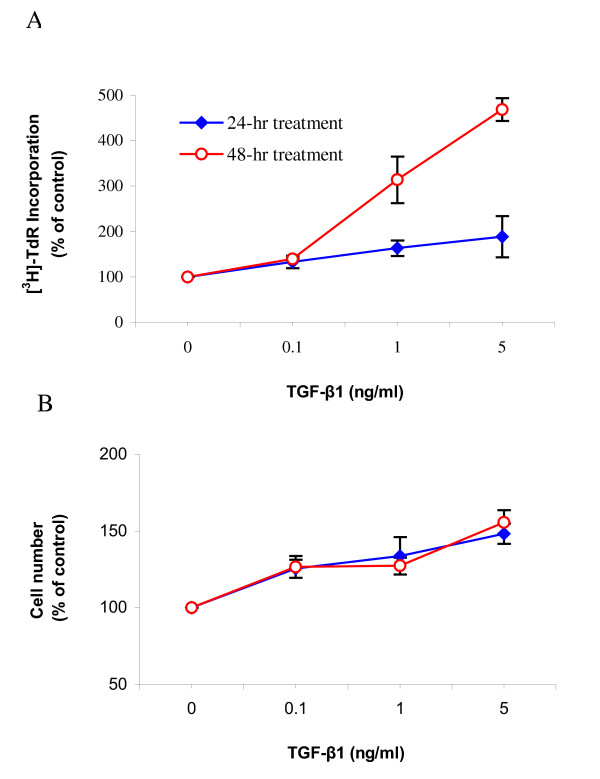
**TGF-β1 concentration-dependently increased proliferation of ASMCs. **Confluent and growth-arrested ASMCs were incubated with various concentrations of TGF-β1 for 24 or 48 hours prior to [^3^H]-thymidine incorporation assay (A) or cell counting (B). Significant differences were detected at all concentrations of TGF-β1 treatment compared to the untreated control, p < 0.05 to p < 0.0001, n = 4–18.

All concentrations of TGF-β1 (0.1, 1 and 5 ng/ml) induced significant increase in [^3^H]-thymidine incorporation by the ASMCs. Incubation of ASMCs with TGF-β1 for 48 hours induced more proliferation than 24 hours of incubation (Figure 2A). The TGF-β1 induced DNA synthesis was blocked by the addition of anti-TGF-β1 antibody (data not shown). TGF-β1 also induced a significant concentration-dependent increase in cell numbers (Figure 2B); however, the magnitude of the increased cell number was lower than the increased [^3^H]-thymidine incorporation, suggesting that as a parameter of cell proliferation, [^3^H]-thymidine incorporation is more sensitive than cell number.

### TGF-β1 augmented serum-induced proliferation

**Figure 3:**
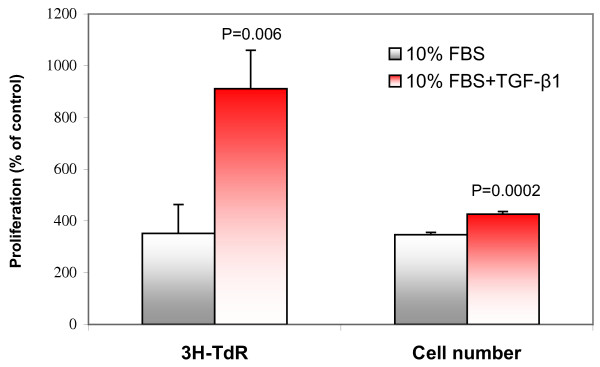
**TGF-β1 enhanced serum-induced proliferation of ASMCs. **Confluent and growth-arrested ASMCs were treated with 10% FBS in the absence or presence of TGF-β1 (1 ng/ml) for 48 hours and the changes of [^3^H]-thymidine incorporation (n = 9) and cell number (n = 6) were determined. All values are % of untreated control cultured in 0.2% BSA/DMEM. p values indicated were compared to control (10% FBS only).

Serum contains a variety of mitogenic substances that may enter the airways as protein exudates during airway inflammation. ASMCs can respond synergistically to a wide variety of mitogen combinations [39]. TGF-β may interact with these substances and affect ASMC proliferation. To determine this, we treated confluent, serum-free ASMCs with 10% FBS in the absence or presence of TGF-β1 (1 ng/ml) for 48 hours and measured the changes of thymidine incorporation and cell number. DNA synthesis and cell number were significantly increased after treatment with 10% FBS compared to the cells cultured in serum-free medium (Figure 3). The serum-induced increases in thymidine incorporation and cell number were further enhanced by addition of 1 ng/ml TGF-β1 (Figure 3). Similar changes, to a lesser extent, were observed when 1% FBS was used (data not shown).

### Roles of MAPKs in TGF-β1 induced proliferation

**Figure 4:**
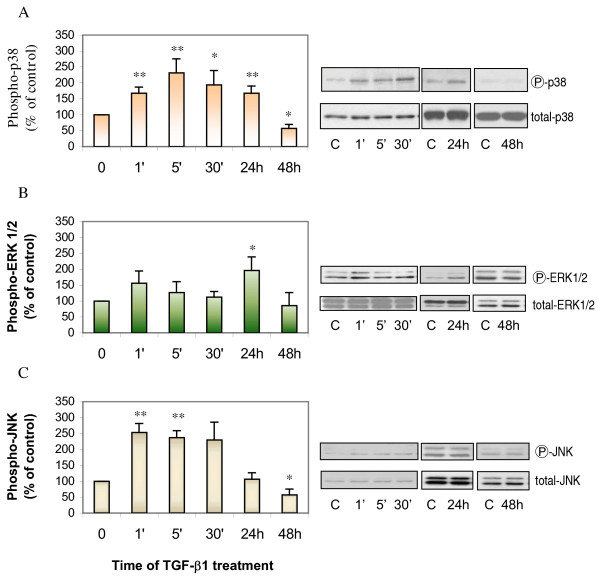
**TGF-β1 increased expression of phosphorylated MAPKs in ASMCs. **Confluent and growth-arrested ASMCs were incubated with 1 ng/ml of TGF-β1 for 1, 5, 30 minutes, 24 or 48 hours prior to protein extraction and Western analysis for phosphorylated or total p38 (Panel A), ERK1/2 (Panel B), and JNK (Panel C). * p < 0.05, ** p < 0.01, ** p < 0.001 compared to control, n = 4–10, C = control.

**Figure 5:**
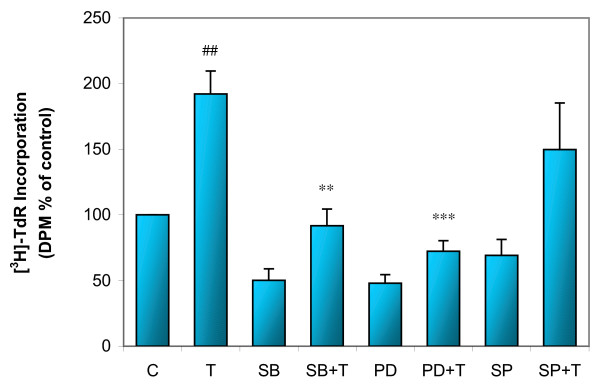
**Effects of MAPKs inhibitors on TGF-β1 induced increase of proliferation in ASMCs. **Confluent and growth-arrested ASMCs were pretreated for 1 hour with SB 203580, PD 98059, or SP 600125, prior to 24-hour treatment with 1 ng/ml of TGF-β1 (T). DNA synthesis was measured by [^3^H]-thymidine incorporation assay. Inhibition of phosphorylated p38 and ERK1/2 reduced TGF-β1 induced DNA synthesis. ## p < 0.01 compared to untreated control (C), ** p < 0.01, *** p < 0.001 compared to T, n = 7–8.

**Figure 6:**
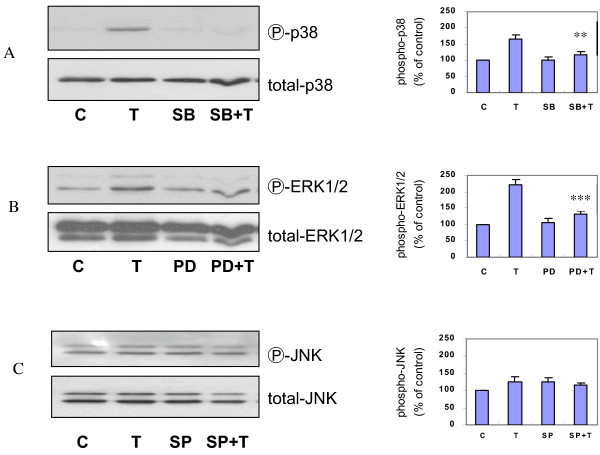
**Effects of MAPKs inhibitors on TGF-β1 induced activation of MAPKs. **Confluent and growth-arrested ASMCs were pretreated for 1 hour with SB 203580, PD 98059, or SP 600125, prior to 24-hour treatment with 1 ng/ml of TGF-β1 (T), followed by protein extraction and Western analysis for phosphorylated or total p38 (Panel A), ERK1/2 (Panel B), and JNK (Panel C). The blots are representatives of 3 independent experiments. C = control. ** p < 0.01 *** p < 0.001 compared to T.

Next, we determined if MAPKs play any role in TGF-β1 induced increase in proliferation. ASMCs were treated with 1 ng/ml of TGF-β1 for 1, 5, 30 minutes, 24 and 48 hours, followed by extraction of the cellular protein. The expressions of total and phosphorylated p38, ERK1/2, and JNK were determined by Western analysis. TGF-β1 induced rapid increases in phospho-p38 (Figure 4A) and phospho-JNK (Figure 4C), beginning as early as 1 minute after addition of TGF-β1 and lasting up to 24 hours for phospho-p38. However, the phosphorylation of JNK was early and brief in duration (Figure 4C). Longer treatment (48 hours) with TGF-β1 led to a decrease in both phospho-p38 and phospho-JNK. The TGF-β1 induced increases in phospho-ERK1/2 occurred only after 24-hour treatment and this was not decreased by 48-hour treatment (Figure 4B). There was no change in the expression of total p38, ERK1/2, and JNK. In addition, to confirm that the TGF-β1 induced induction of phosphorylated p38, JNK, or ERK1/2 regulated cell proliferation, ASMCs were pretreated for one hour with SB 203580, PD 98059, or SP 600125, followed by 24-hour TGF-β1 treatment and [^3^H]-thymidine incorporation assay. The TGF-β1 induced DNA synthesis was attenuated by SB 203580 or PD 98059, but not SP 600125 (Figure 5). Furthermore, total and phosphorylated p38, ERK1/2, and JNK were determined using the cellular protein of ASMCs treated with TGF-β1 for 24 hours in the presence or absence of SB 203580, PD 98059, or SP 600125. Western analysis revealed that TGF-β1 induced phosphorylation of p38 and ERK1/2 were inhibited by SB 203580, PD 98059, respectively (Figure 6). There were no changes in phosphorylation of JNK between cells of control, TGF-β1, and SP 600125 plus TGF-β1 treatment (Figure 6). These data suggest that TGF-β1 induced increase in proliferation may be mediated by the activation of p38 and ERK1/2.

### Roles of FGF-2, PDGF, EGF and IGF-I in TGF-β1 induced proliferation

**Figure 7:**
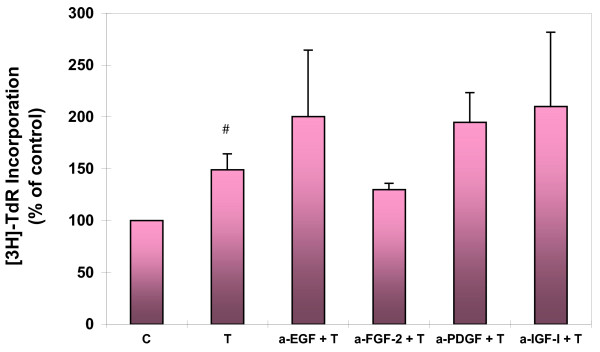
**Role of EGF, FGF-2, PDGF and IGF-I in TGF-β1 induced proliferation of ASMCs. **Confluent and growth-arrested ASMCs were treated with TGF-β1 (1 ng/ml) in the absence or presence of neutralizing antibodies to EGF, FGF-2, PDGF and IGF-I for 48 hours prior to [^3^H]-thymidine incorporation assay. # p < 0.01, compared to untreated control (C). There were no significant differences (p > 0.05) in the DNA synthesis between TGF-β1 treated cells with and without pretreatment with these antibodies.

To examine if the TGF-β1 induced proliferation of ASMCs is a secondary effect mediated by other growth factors that had been reported to be induced by TGF-β1 [20,23,40-42], ASMCs were treated with TGF-β1 in the absence or presence of neutralizing antibodies against FGF-2, PDGF, EGF, and IGF-I (all from R&D Systems). [^3^H]-thymidine incorporation was performed after 48-hour treatment with TGF-β1. As shown in Figure 7, there were no significant differences in the DNA synthesis between TGF-β1 treated ASMCs with and without these antibodies. The data suggest that TGF-β1 induced ASMC proliferation may not be mediated by these previously described TGF-β1 inducible growth factors.

## Discussion

In this study we have demonstrated that TGF-β1 increases proliferation in serum-free condition and enhances serum-induced proliferation of confluent ASMCs. This observation is consistent with the reports of others in which confluent ASMCs were treated with TGF-β1 in the presence of 0.5 – 5% FBS [24,26,43]. These findings have important clinical significance, because over expression of TGF-β1 mRNA and protein was found in bronchial biopsies from severe and moderate asthmatics [5,7,44,45]. In addition, it was reported that basal TGF-β1 levels in the airways were elevated in atopic asthma and that these levels increased further in response to allergen exposure [6]. Most recently, it was found that C-509T SNP of the TGF-β1 gene is an important susceptibility locus for asthma [46]. Our previous data also demonstrated that wounded ASMCs released biologically active TGF-β1, which in turn induced collagen and fibronectin synthesis [11,12]. Therefore, it is conceivable that in chronic asthmatics with repeated episode of injury and inflammation, TGF-β1 is synthesized and released into the airways or within the smooth muscle cells of the airways. The release and persistent presence of TGF-β1 in asthmatic airways may gradually induce airway smooth muscle hypertrophy and hyperplasia. Moreover, our finding that TGF-β1 enhances serum-induced ASMC proliferation may occur in asthmatic airways where there is inflammation leading to increase in vascular permeability and leakage of plasma that contains cytokines mitogenic for ASMCs. Our results suggest that the mitogenic effects of the cytokines would be enhanced by TGF-β1, and augment the ASMC hyperplasia and remodeling changes. The proliferative changes, combined with TGF-β1 induced connective tissue synthesis in ASMCs [11,12,14], would thicken the airway wall, reduce baseline airway caliber and exaggerate airway narrowing. Unlike Black and co-workers' finding that TGF-β1 treatment for 24 hours and 48 hours led to inhibition and promotion, respectively, of ASMC growth, in our present study, both 24-hour and 48-hour treatment with TGF-β1 induced increases in ASMC proliferation. The difference for the cell response after 24-hour TGF-β1 treatment may be due to the different culture condition. Black et al treated ASMCs in the presence of 2% serum in the culture medium, while we did not use any serum when we treated the cells. Therefore, the different extent of serum-deprivation may affect the cell response to mitogens.

Little is known about the mechanisms by which TGF-β1 affects ASMC proliferation. In human ASMCs, it was found that TGF-β1 induced a 10–20 fold increase in insulin-like growth factor binding protein-3 (IGFBP-3) mRNA and protein and a 2-fold increase in cell proliferation, which was blocked by IGFBP-3 antisense or IGFBP-3 neutralizing antibody, suggesting IGFBP-3 mediates TGF-β1 induced proliferation [43]. In cells other than ASMCs, it was suggested that release of PDGF mediated by TGF-β1 induces mesenchymal cells proliferation [20,42,23]. For example, Battegay and co-workers found that TGF-β1 induced human dermal fibroblasts, chondrocytes, and arterial smooth muscle cell proliferation at low concentrations by stimulating autocrine PDGF-AA secretion [20]. Other studies showed that TGF-β1 induced marked growth responses, alone or in combination with EGF, FGF-2, or PDGF-BB, that were largely independent of PDGF-AA [41]. We had recently demonstrated that treatment of primary interstitial pulmonary fibroblasts with TGF-β1 released large quantity of FGF-2, which led to proliferation. This TGF-β1 induced proliferation of the fibroblasts was mediated by FGF-2, but not EGF, IGF-I or PDGF [[47] and our unpublished data). In our present study, we used neutralizing antibodies against EGF, FGF-2, IGF-I or PDGF to examine the possible role of these growth factors in TGF-β1 induced ASMC proliferation. However, these antibodies did not block the TGF-β1 induced DNA synthesis. Our data suggest that the TGF-β1 induced proliferation of ASMCs in our model might be independent of the growth factors previously reported to mediate the proliferative effects of TGF-β1 in mesenchymal cells.

Phosphorylation of ERK1/2 has been reported to mediate mitogen-induced proliferation, while the phosphorylation of JNK and p38 are activated by a variety of non-specific stimuli such as changes in oxidation, osmolarity, and inflammatory cytokines [28,48]. The important roles of MAPKs activation in ASMC proliferation induced by endothelin-1, thrombin, FGF-2, PDGF, EGF, IGF-I, 5-HT and so on have been reported [29,49-37]. However, it is not known if MAPKs mediate TGF-β1 induced ASMC proliferation. In this study, for the first time, we have demonstrated that TGF-β1 induced proliferation of ASMCs is associated with increased expression of phosphorylated ERK1/2, p38, and JNK with different kinetics of induction. Since the inhibitors of p38 and ERK blocked TGF-β1 induced proliferation, our data suggest that the activation of p38 and ERK is important for the TGF-β1 induced increase in ASMC proliferation. Our results are partly supported by another study using tracheal smooth muscle cells, which demonstrated that activation of p38 pathway by TGF-β modulated smooth muscle migration and remodeling [50]. In our study, there are some differences in the time required for activation of MAPKs after TGF-β1 stimulation amongst the 3 MAPKs. P38 and JNK were rapidly activated by TGF-β1, which was as early as 1 minute. However, the activation of ERK1/2 required prolonged treatment with TGF-β1 (24 hours). The activation of JNK lasted only 5 min, and the blockade of JNK activation failed to inhibit the ASMC proliferation induced by 24-hour of TGF-β1 treatment, indicating that the activation of JNK may not be important in mediating TGF-β1 induced proliferation of ASMCs. Interestingly, our finding is similar to a previous report using human lung fibroblasts, in which TGF-β1 activated ERK and p38 but not JNK [40]. The authors used 30-minute, 2-, 6-, 16-, and 24-hour TGF-β1 treatment and found that phosphorylation of p38 began within 30 minutes, while ERK1/2 activation began at 2 hour with maximal induction by 16 hour. They also found that activator protein-1(AP-1) binding depended on ERK1/2 but not p38 activation. However, using fibroblasts, we and others reported that TGF-β1 activated JNK and p38, but not ERK1/2 [47,51]. In another study, an interaction between ERK and p38 in macrophages was proposed in which TGF-β1 activated ERK, which in turn up-regulated MAPK phosphatase-1, thereby inactivating p38 [52]. A recent study using selective inhibitors of the three MAPKs [53] showed that inhibition of one of the intracellular pathway was sufficient to inhibit IL-1β induced ASMC proliferation and simultaneous inhibition did not lead to further reduction in the proliferation, suggesting the three major MAPK pathways are independent regulators of IL-1β dependent proliferation of rat ASMCs. Taken together, the above data indicate that one or more MAPK can be activated by TGF-β1 and the different MAPKs may act through different pathways in TGF-β1 induced proliferation of mesenchymal cells.

Our findings differ from a study by Cohen et al [54] in which TGF-β1 alone had no effect on human ASMC proliferation, but TGF-β1 inhibited EGF- and thrombin-induced DNA synthesis, which was independent of ERK activation. However, it is somewhat incomparable with our data, because in addition to the species difference, the cells they used had no proliferative response to TGF-β1 alone, and they did not show whether TGF-β1 affected the activation of MAPKs. In addition, they used 5 μg/ml of insulin in their serum-free medium, which may affect the cell's response to growth factors or downstream mediators.

The effects of TGF-β are mediated by TβR I and TβR II, which phosphorylate Smad 2 and Smad 3. The phosphorylated Smad 2 and Smad 3 bind Smad 4. The resultant complex translocates to the nucleus and activates the expression of target genes. It was demonstrated that Ras/MEK/ERK pathway is partially required in order for TGF-β to activate Smad 55, and is also required for the Smad-mediated induction of connective tissue growth factor (CTGF) by TGF-β2 56. In addition, it was reported that constitutive activation of p38 pathway-induced transcriptional activation was enhanced synergistically by coexpression of Smad2 and Smad 4, and was inhibited by expression of C-terminal truncated, dominant negative Smad 4 57. Zhang and coworkers demonstrated a direct interaction between Smad 3/4 and two transcriptional factors (c-Jun and c-Fos) among the targets of the MARK pathways 58. Most recently, in cultured airway smooth muscle cells, Xie and coworkers 59 found that TGF-β1 induced a significant activation of Smad 2/3 and translocation of phospho-Smad 2/3 and Smad 4 from cytosol to nucleus, as well as a time- and concentration-dependent expression of CTGF gene and protein. The TGF-β1 induced phosphorylation of Smad 2/3 and the expression of CTGF mRNA and protein were all blocked by the inhibition of ERK and JNK, but not by the inhibition of p38 and phosphatidylinositol 3-kinase (PI3K). The evidences given emphasize that there is a stimulatory interaction between MAPK pathway and Smad pathway in the context of TGF-β signaling. This interaction may play an important role in the airway remodeling. For example, CTGF is a downstream mediator of TGF-β fibrotic effects and is constitutively overexpressed in fibrotic airways. It is not clear whether this interaction is involved in the ASMC proliferation, however, it is possible in our present work that the TGF-β1 induced expression of MAPKs cross-talks with Smad pathway, and they act together which results in proliferation and fibrosis.

## Conclusion

In conclusion, our results demonstrate that TGF-β1 increases ASMC proliferation, and also enhances serum-induced ASMC proliferation. In addition, the activation of p38 and ERK play an important role in mediating the TGF-β1 induced proliferation by ASMCs. These findings suggest that TGF-β1 which is expressed in airways of asthmatics may contribute to irreversible airway remodeling by enhancing ASMC proliferation.

## Competing interests

The author(s) declare that they have no competing interests.

## Authors' contributions

GC carried out all the experiments, wrote the manuscript and helped with the intellectual development of the work. NK obtained funding for the work, initiated and supported the intellectual development of the work.
